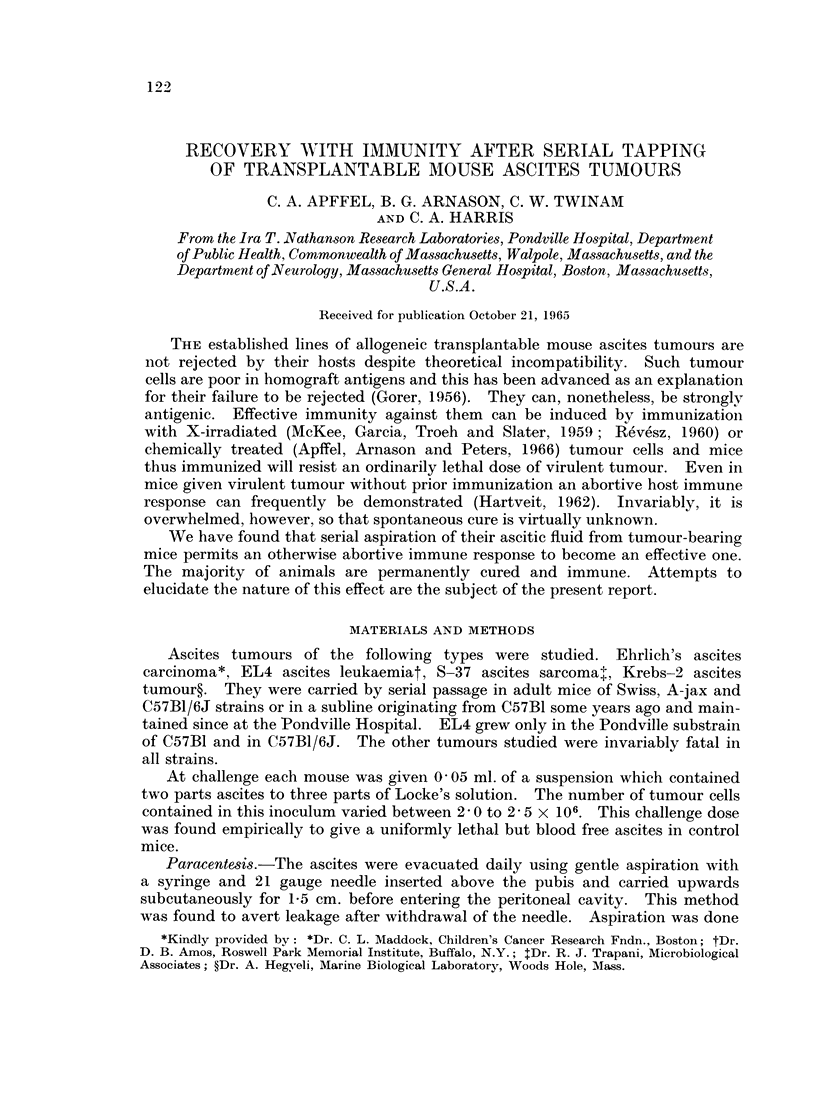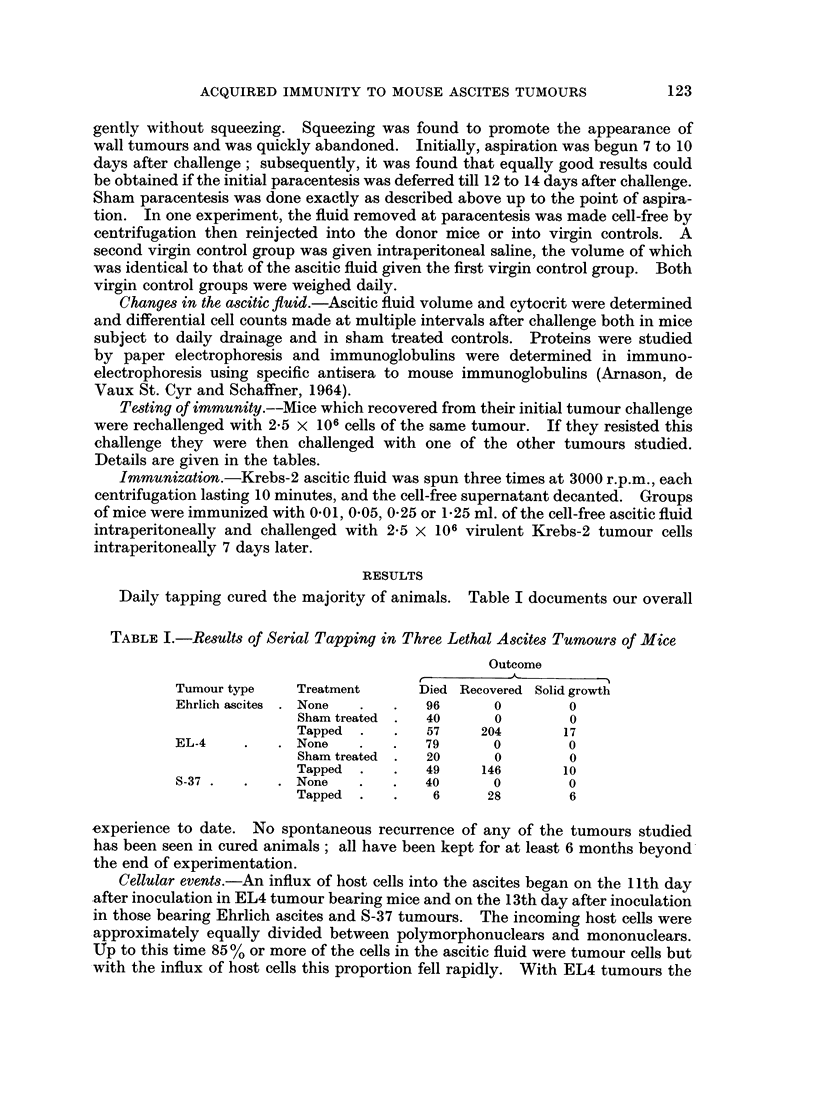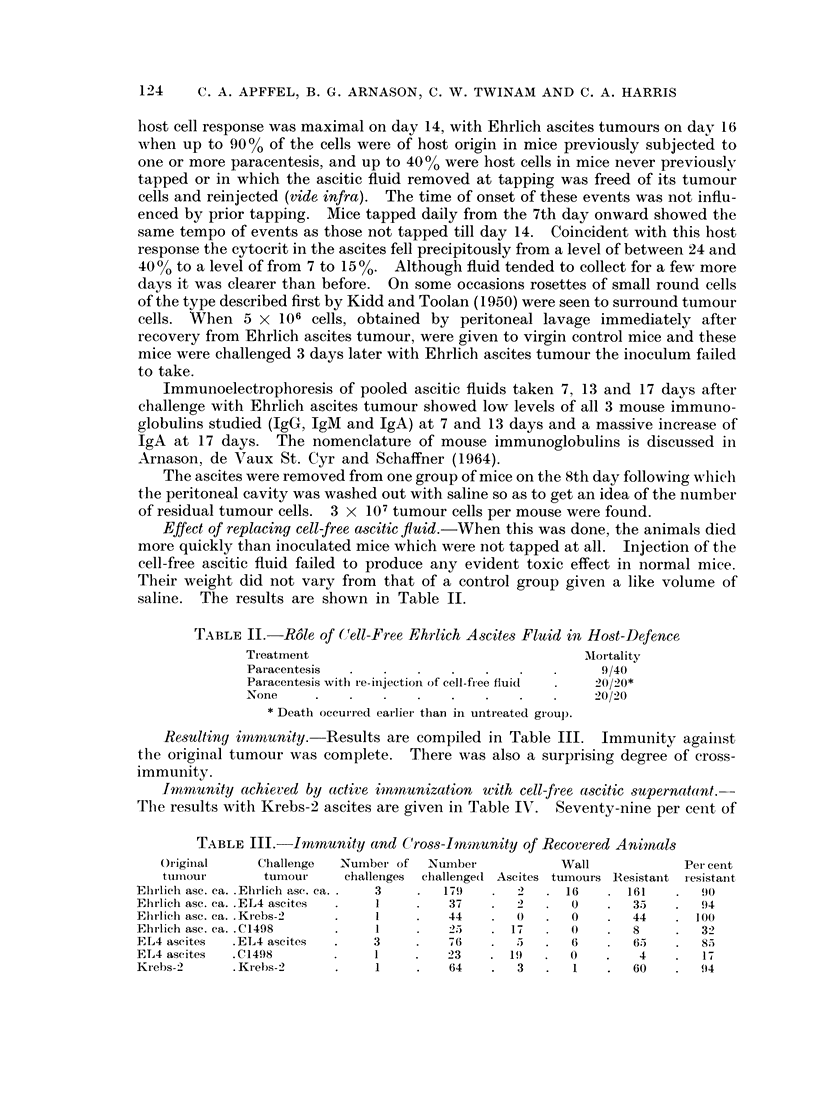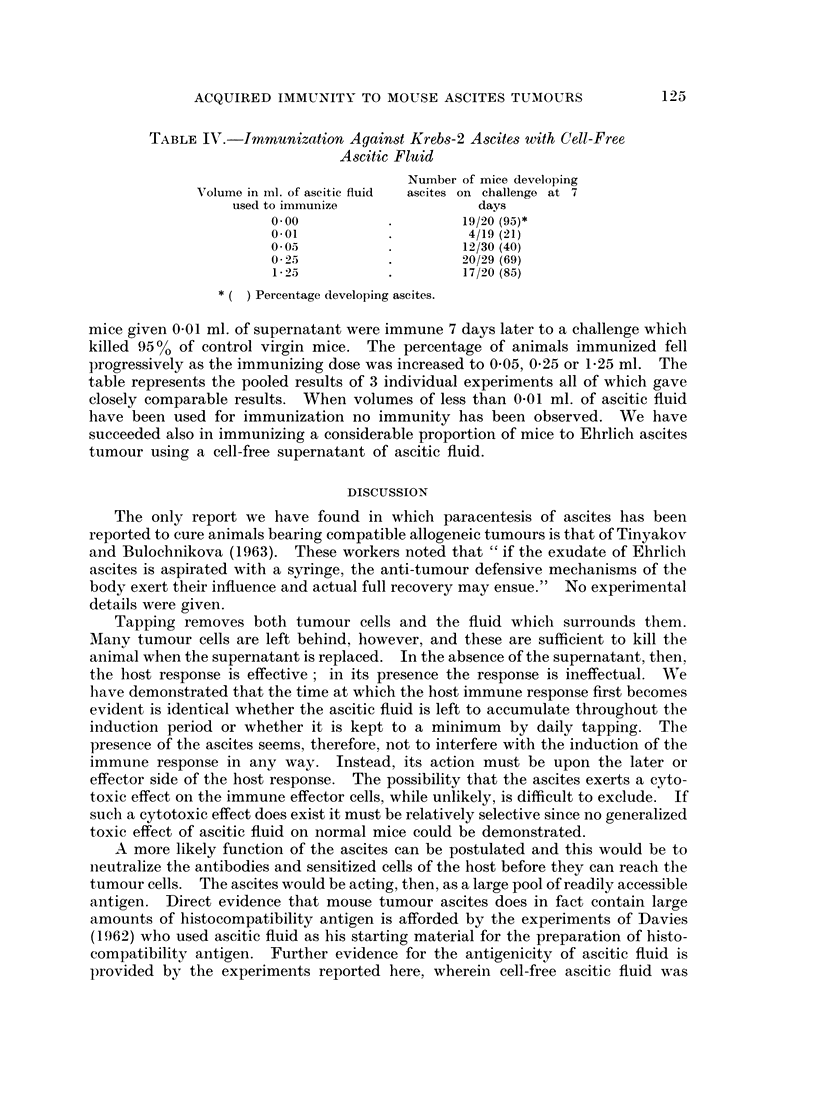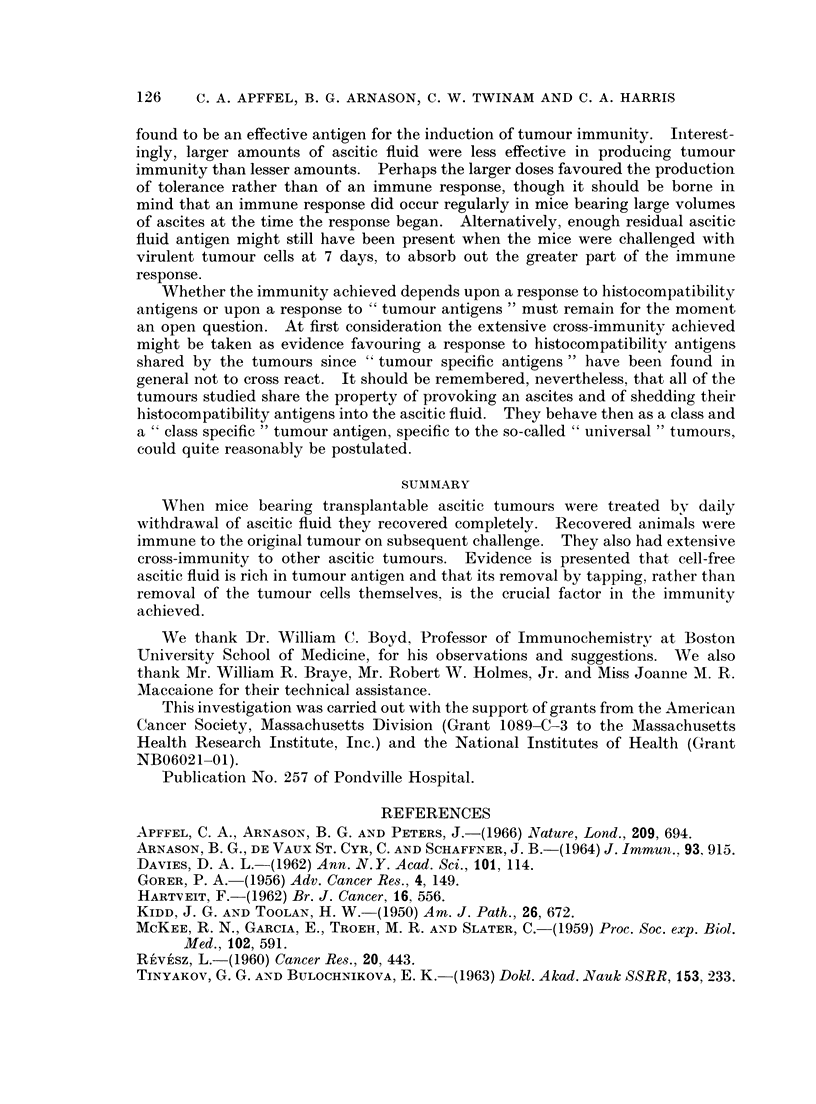# Recovery with immunity after serial tapping of transplantable mouse ascites tumours.

**DOI:** 10.1038/bjc.1966.13

**Published:** 1966-03

**Authors:** C. A. Apffel, B. G. Arnason, C. W. Twinam, C. A. Harris


### RECOVERY WITH IMMUNITY AFTER SERIAL TAPPING OF TRANSPLANTABLE MOUSE ASCITES TUMOURS

C. A. APFFEL, B. G. ARNASON, C. W. TWINAM AND C. A. HARRIS

*From the Ira T. Nathanson Research Laboratories, Pondville Hospital, Department of Public Health, Commonwealth of Massachusetts, Walpole, Massachusetts, and the Department of Neurology, Massachusetts General Hospital, Boston, Massachusetts, U.S.A.*

Received for publication October 21, 1965

THE established lines of allogeneic transplantable mouse ascites tumours are not rejected by their hosts despite theoretical incompatibility. Such tumour cells are poor in homograft antigens and this has been advanced as an explanation for their failure to be rejected (Gorer, 1956). They can, nonetheless, be strongly antigenic. Effective immunity against them can be induced by immunization with X-irradiated (McKee, Garcia, Troeh and Slater, 1959; Révész, 1960) or chemically treated (Apffel, Arnason and Peters, 1966) tumour cells and mice thus immunized will resist an ordinarily lethal dose of virulent tumour. Even in mice given virulent tumour without prior immunization an abortive host immune response can frequently be demonstrated (Hartveit, 1962). Invariably, it is overwhelmed, however, so that spontaneous cure is virtually unknown.

We have found that serial aspiration of their ascitic fluid from tumour-bearing mice permits an otherwise abortive immune response to become an effective one. The majority of animals are permanently cured and immune. Attempts to elucidate the nature of this effect are the subject of the present report.

### MATERIALS AND METHODS

Ascites tumours of the following types were studied. Ehrlich's ascites carcinoma*, EL4 ascites leukaemia†, S-37 ascites sarcoma‡, Krebs-2 ascites tumour§. They were carried by serial passage in adult mice of Swiss, A-jax and C57Bl/6J strains or in a subline originating from C57Bl some years ago and maintained since at the Pondville Hospital. EL4 grew only in the Pondville substrain of C57Bl and in C57Bl/6J. The other tumours studied were invariably fatal in all strains.

At challenge each mouse was given 0·05 ml. of a suspension which contained two parts ascites to three parts of Locke's solution. The number of tumour cells contained in this inoculum varied between 2·0 to 2·5 $\times 10^6$. This challenge dose was found empirically to give a uniformly lethal but blood free ascites in control mice.

*Paracentesis.*—The ascites were evacuated daily using gentle aspiration with a syringe and 21 gauge needle inserted above the pubis and carried upwards subcutaneously for 1·5 cm. before entering the peritoneal cavity. This method was found to avert leakage after withdrawal of the needle. Aspiration was done

*Kindly provided by: *Dr. C. L. Maddock, Children's Cancer Research Fndn., Boston; †Dr. D. B. Amos, Roswell Park Memorial Institute, Buffalo, N.Y.; ‡Dr. R. J. Trapani, Microbiological Associates; §Dr. A. Hegyeli, Marine Biological Laboratory, Woods Hole, Mass.

gently without squeezing. Squeezing was found to promote the appearance of wall tumours and was quickly abandoned. Initially, aspiration was begun 7 to 10 days after challenge ; subsequently, it was found that equally good results could be obtained if the initial paracentesis was deferred till 12 to 14 days after challenge. Sham paracentesis was done exactly as described above up to the point of aspiration. In one experiment, the fluid removed at paracentesis was made cell-free by centrifugation then reinjected into the donor mice or into virgin controls. A second virgin control group was given intraperitoneal saline, the volume of which was identical to that of the ascitic fluid given the first virgin control group. Both virgin control groups were weighed daily.

*Changes in the ascitic fluid.*—Ascitic fluid volume and cytocrit were determined and differential cell counts made at multiple intervals after challenge both in mice subject to daily drainage and in sham treated controls. Proteins were studied by paper electrophoresis and immunoglobulins were determined in immunoelectrophoresis using specific antisera to mouse immunoglobulins (Arnason, de Vaux St. Cyr and Schaffner, 1964).

*Testing of immunity.*—Mice which recovered from their initial tumour challenge were rechallenged with $2{\cdot}5 \times 10^6$ cells of the same tumour. If they resisted this challenge they were then challenged with one of the other tumours studied. Details are given in the tables.

*Immunization.*—Krebs-2 ascitic fluid was spun three times at 3000 r.p.m., each centrifugation lasting 10 minutes, and the cell-free supernatant decanted. Groups of mice were immunized with 0·01, 0·05, 0·25 or 1·25 ml. of the cell-free ascitic fluid intraperitoneally and challenged with $2{\cdot}5 \times 10^6$ virulent Krebs-2 tumour cells intraperitoneally 7 days later.

### RESULTS

Daily tapping cured the majority of animals. Table I documents our overall

TABLE I.—*Results of Serial Tapping in Three Lethal Ascites Tumours of Mice*

| | | Outcome | | |
|---|---|---|---|---|
| Tumour type | Treatment | Died | Recovered | Solid growth |
| Ehrlich ascites | None | 96 | 0 | 0 |
| | Sham treated | 40 | 0 | 0 |
| | Tapped | 57 | 204 | 17 |
| EL-4 | None | 79 | 0 | 0 |
| | Sham treated | 20 | 0 | 0 |
| | Tapped | 49 | 146 | 10 |
| S-37 | None | 40 | 0 | 0 |
| | Tapped | 6 | 28 | 6 |

experience to date. No spontaneous recurrence of any of the tumours studied has been seen in cured animals ; all have been kept for at least 6 months beyond the end of experimentation.

*Cellular events.*—An influx of host cells into the ascites began on the 11th day after inoculation in EL4 tumour bearing mice and on the 13th day after inoculation in those bearing Ehrlich ascites and S-37 tumours. The incoming host cells were approximately equally divided between polymorphonuclears and mononuclears. Up to this time 85% or more of the cells in the ascitic fluid were tumour cells but with the influx of host cells this proportion fell rapidly. With EL4 tumours the host cell response was maximal on day 14, with Ehrlich ascites tumours on day 16 when up to 90% of the cells were of host origin in mice previously subjected to one or more paracentesis, and up to 40% were host cells in mice never previously tapped or in which the ascitic fluid removed at tapping was freed of its tumour cells and reinjected (*vide infra*). The time of onset of these events was not influenced by prior tapping. Mice tapped daily from the 7th day onward showed the same tempo of events as those not tapped till day 14. Coincident with this host response the cytocrit in the ascites fell precipitously from a level of between 24 and 40% to a level of from 7 to 15%. Although fluid tended to collect for a few more days it was clearer than before. On some occasions rosettes of small round cells of the type described first by Kidd and Toolan (1950) were seen to surround tumour cells. When $5 \times 10^6$ cells, obtained by peritoneal lavage immediately after recovery from Ehrlich ascites tumour, were given to virgin control mice and these mice were challenged 3 days later with Ehrlich ascites tumour the inoculum failed to take.

Immunoelectrophoresis of pooled ascitic fluids taken 7, 13 and 17 days after challenge with Ehrlich ascites tumour showed low levels of all 3 mouse immunoglobulins studied (IgG, IgM and IgA) at 7 and 13 days and a massive increase of IgA at 17 days. The nomenclature of mouse immunoglobulins is discussed in Arnason, de Vaux St. Cyr and Schaffner (1964).

The ascites were removed from one group of mice on the 8th day following which the peritoneal cavity was washed out with saline so as to get an idea of the number of residual tumour cells. $3 \times 10^7$ tumour cells per mouse were found.

*Effect of replacing cell-free ascitic fluid.*—When this was done, the animals died more quickly than inoculated mice which were not tapped at all. Injection of the cell-free ascitic fluid failed to produce any evident toxic effect in normal mice. Their weight did not vary from that of a control group given a like volume of saline. The results are shown in Table II.

TABLE II.—*Rôle of Cell-Free Ehrlich Ascites Fluid in Host-Defence*

| Treatment | Mortality |
|---|---|
| Paracentesis | 9/40 |
| Paracentesis with re-injection of cell-free fluid | 20/20* |
| None | 20/20 |

* Death occurred earlier than in untreated group.

*Resulting immunity.*—Results are compiled in Table III. Immunity against the original tumour was complete. There was also a surprising degree of cross-immunity.

*Immunity achieved by active immunization with cell-free ascitic supernatant.*—The results with Krebs-2 ascites are given in Table IV. Seventy-nine per cent of

TABLE III.—*Immunity and Cross-Immunity of Recovered Animals*

| Original tumour | Challenge tumour | Number of challenges | Number challenged | Ascites | Wall tumours | Resistant | Per cent resistant |
|---|---|---|---|---|---|---|---|
| Ehrlich asc. ca. | Ehrlich asc. ca. | 3 | 179 | 2 | 16 | 161 | 90 |
| Ehrlich asc. ca. | EL4 ascites | 1 | 37 | 2 | 0 | 35 | 94 |
| Ehrlich asc. ca. | Krebs-2 | 1 | 44 | 0 | 0 | 44 | 100 |
| Ehrlich asc. ca. | C1498 | 1 | 25 | 17 | 0 | 8 | 32 |
| EL4 ascites | EL4 ascites | 3 | 76 | 5 | 6 | 65 | 85 |
| EL4 ascites | C1498 | 1 | 23 | 19 | 0 | 4 | 17 |
| Krebs-2 | Krebs-2 | 1 | 64 | 3 | 1 | 60 | 94 |

TABLE IV.—*Immunization Against Krebs-2 Ascites with Cell-Free Ascitic Fluid*

| Volume in ml. of ascitic fluid used to immunize | | Number of mice developing ascites on challenge at 7 days |
|---|---|---|
| 0·00 | . | 19/20 (95)* |
| 0·01 | . | 4/19 (21) |
| 0·05 | . | 12/30 (40) |
| 0·25 | . | 20/29 (69) |
| 1·25 | . | 17/20 (85) |

* ( ) Percentage developing ascites.

mice given 0·01 ml. of supernatant were immune 7 days later to a challenge which killed 95% of control virgin mice. The percentage of animals immunized fell progressively as the immunizing dose was increased to 0·05, 0·25 or 1·25 ml. The table represents the pooled results of 3 individual experiments all of which gave closely comparable results. When volumes of less than 0·01 ml. of ascitic fluid have been used for immunization no immunity has been observed. We have succeeded also in immunizing a considerable proportion of mice to Ehrlich ascites tumour using a cell-free supernatant of ascitic fluid.

### DISCUSSION

The only report we have found in which paracentesis of ascites has been reported to cure animals bearing compatible allogeneic tumours is that of Tinyakov and Bulochnikova (1963). These workers noted that " if the exudate of Ehrlich ascites is aspirated with a syringe, the anti-tumour defensive mechanisms of the body exert their influence and actual full recovery may ensue." No experimental details were given.

Tapping removes both tumour cells and the fluid which surrounds them. Many tumour cells are left behind, however, and these are sufficient to kill the animal when the supernatant is replaced. In the absence of the supernatant, then, the host response is effective ; in its presence the response is ineffectual. We have demonstrated that the time at which the host immune response first becomes evident is identical whether the ascitic fluid is left to accumulate throughout the induction period or whether it is kept to a minimum by daily tapping. The presence of the ascites seems, therefore, not to interfere with the induction of the immune response in any way. Instead, its action must be upon the later or effector side of the host response. The possibility that the ascites exerts a cytotoxic effect on the immune effector cells, while unlikely, is difficult to exclude. If such a cytotoxic effect does exist it must be relatively selective since no generalized toxic effect of ascitic fluid on normal mice could be demonstrated.

A more likely function of the ascites can be postulated and this would be to neutralize the antibodies and sensitized cells of the host before they can reach the tumour cells. The ascites would be acting, then, as a large pool of readily accessible antigen. Direct evidence that mouse tumour ascites does in fact contain large amounts of histocompatibility antigen is afforded by the experiments of Davies (1962) who used ascitic fluid as his starting material for the preparation of histocompatibility antigen. Further evidence for the antigenicity of ascitic fluid is provided by the experiments reported here, wherein cell-free ascitic fluid was found to be an effective antigen for the induction of tumour immunity. Interestingly, larger amounts of ascitic fluid were less effective in producing tumour immunity than lesser amounts. Perhaps the larger doses favoured the production of tolerance rather than of an immune response, though it should be borne in mind that an immune response did occur regularly in mice bearing large volumes of ascites at the time the response began. Alternatively, enough residual ascitic fluid antigen might still have been present when the mice were challenged with virulent tumour cells at 7 days, to absorb out the greater part of the immune response.

Whether the immunity achieved depends upon a response to histocompatibility antigens or upon a response to " tumour antigens " must remain for the moment an open question. At first consideration the extensive cross-immunity achieved might be taken as evidence favouring a response to histocompatibility antigens shared by the tumours since " tumour specific antigens " have been found in general not to cross react. It should be remembered, nevertheless, that all of the tumours studied share the property of provoking an ascites and of shedding their histocompatibility antigens into the ascitic fluid. They behave then as a class and a " class specific " tumour antigen, specific to the so-called " universal " tumours, could quite reasonably be postulated.

### SUMMARY

When mice bearing transplantable ascitic tumours were treated by daily withdrawal of ascitic fluid they recovered completely. Recovered animals were immune to the original tumour on subsequent challenge. They also had extensive cross-immunity to other ascitic tumours. Evidence is presented that cell-free ascitic fluid is rich in tumour antigen and that its removal by tapping, rather than removal of the tumour cells themselves, is the crucial factor in the immunity achieved.

We thank Dr. William C. Boyd, Professor of Immunochemistry at Boston University School of Medicine, for his observations and suggestions. We also thank Mr. William R. Braye, Mr. Robert W. Holmes, Jr. and Miss Joanne M. R. Maccaione for their technical assistance.

This investigation was carried out with the support of grants from the American Cancer Society, Massachusetts Division (Grant 1089–C–3 to the Massachusetts Health Research Institute, Inc.) and the National Institutes of Health (Grant NB06021–01).

Publication No. 257 of Pondville Hospital.

### REFERENCES

Apffel, C. A., Arnason, B. G. and Peters, J.—(1966) *Nature, Lond.*, **209**, 694.
Arnason, B. G., de Vaux St. Cyr, C. and Schaffner, J. B.—(1964) *J. Immun.*, **93**, 915.
Davies, D. A. L.—(1962) *Ann. N.Y. Acad. Sci.*, **101**, 114.
Gorer, P. A.—(1956) *Adv. Cancer Res.*, **4**, 149.
Hartveit, F.—(1962) *Br. J. Cancer*, **16**, 556.
Kidd, J. G. and Toolan, H. W.—(1950) *Am. J. Path.*, **26**, 672.
McKee, R. N., Garcia, E., Troeh, M. R. and Slater, C.—(1959) *Proc. Soc. exp. Biol. Med.*, **102**, 591.
Révész, L.—(1960) *Cancer Res.*, **20**, 443.
Tinyakov, G. G. and Bulochnikova, E. K.—(1963) *Dokl. Akad. Nauk SSRR*, **153**, 233.